# Strategies for promoting male partner involvement in maternal, newborn and child health care in Kiambu County

**DOI:** 10.11604/pamj.2023.46.102.40935

**Published:** 2023-12-12

**Authors:** Joseph Mukobe Okwako, Grace Wambura Mbuthia, Karani Magutah

**Affiliations:** 1 Department of Midwifery, Jomo Kenyatta University of Agriculture and Technology, Juja, Kenya,; 2 Department of Community Health Nursing, Jomo Kenyatta University of Agriculture and Technology, Juja, Kenya,; 3 Department of Medical Physiology, Moi University, Eldoret, Kenya

**Keywords:** Male partner involvement, accompaniment, determinants, Interventions, strategies, maternal, neonatal and child health

## Abstract

**Introduction:**

the 1994 International Conference on Population and Development (ICPD) recommended that men should share responsibility and be actively involved in sexual and reproductive health. The level of male partner involvement in Kenya remains low despite growing evidence showing its benefits in maternal and newborn health. This study sought to explore strategies for encouraging male partners to accompany their spouses to Maternal and Child Health (MCH) department.

**Methods:**

a qualitative exploratory study was used to gather the views of nurse-midwives and invited men on mechanisms to encourage men to accompany their spouses to MCH clinic. Three and two focused group discussions (FGDs) composed of six to eight participants from nurse-midwives and men were conducted respectively. The FGDs were audio recorded and lasted 60-90 minutes. Content analysis was used to generate themes using MAXQDA 2022 software.

**Results:**

seven categories emerged as strategies that can encourage male partner participation in the Maternal Neonatal and Child Health (MNCH) services: creating community awareness and sensitization, engaging men in the MCH department, reducing waiting time, provision of health services that address male health needs, inviting male partners to the MCH clinic, encouraging MCH staff to be receptive to couples and re-scheduling of MCH working days as well as extension of hours.

**Conclusion:**

evidence-based strategies that adopts multi-level interventions with greater focus on community sensitization and re-organization of the MCH services are important in encouraging men accompany their spouses to clinic and actively participate in the MNCH.

## Introduction

Male partner involvement can be defined as men escorting their wives to Antenatal Care (ANC) and birth, joint decision-making on where to deliver and participation in educational sessions to enhance their knowledge on danger signs in pregnancy, childbirth and the postpartum period as well as helping their partners be prepared for birth and complication readiness [1].

It is believed that men as heads of the family control resources, consult soothsayers to determine the health seeking or treatment for pregnant women, and serve as the final authority on where and when pregnant women should seek medical care [2]. Beyond that, they have no expectation of any further role during ANC and therefore find it unnecessary to attend clinics with their partners. When men accompany their partners to health facilities, it helps them understand their maternal health problems and needs, and eventually, results to greater understanding of their families and the community in general [3]. Despite glaring evidence on the benefits of male partner involvement in maternal, neonatal and child health (MNCH) services, the level of male partner involvement remains low in most African Countries [4-8].

In order to promote male partner involvement, nurses and other healthcare providers need to motivate them by ensuring that they recognize the importance of active involvement in maternal and newborn health services [3]. To achieve this, there is need develop innovative ways such as training healthcare providers on customer care, providing reproductive health information of their spouses, and giving education on health needs and specific roles men play in pregnancy and childbirths [3]. A number of strategies have been employed to booster male participation in MNCH services. Sensitization has been achieved through facility-based couples' education at antenatal care, community-based education for men and women, workplace-based education for expectant fathers, family and community education and social mobilisation campaigns targeting husbands, midwives, and other community members on the roles of men in MNCH services [9]. Other strategies that have been successfully utilized include male incentivization, denying antenatal services to unaccompanied women, and giving priority to couples [9]. Whereas some of the strategies resulted in increased number of men accompanying their spouses, in certain circumstances, it produced unintended outcomes such as low uptake of antenatal services, unfair treatment of women without spouses as well as lack of sustainability. Assessing feasibility and acceptability of context-appropriate strategies for engaging fathers is useful in addressing concerns regarding challenges to engaging fathers. Considering the different sociocultural contexts of previous studies, this study therefore sought to determine strategies required to promote male partner accompaniment and participation in MNCH in a selected public health facility in Kenya.

## Methods

**Study design:** the study adopted an exploratory qualitative design to explore strategies for promoting male partner accompaniment and participation in MNCH services in a Kenyan population.

**Study setting:** the study was conducted among nurse-midwives working in MCH department of Thika and Kiambu Level 5 Hospitals located in Kiambu County, Kenya. The two facilities are County teaching and referral hospitals offering curative, preventive, rehabilitative and preventive health services.

**Participants, sampling and selection:** nurse-midwives and invited male partners of women attending MCH care services were recruited into the study using purposive sampling method because they shared common characteristics under the study. In this case, the nurses had shared expert experience in the study topic while the men had their spouses attending ANC. Upon consenting to participate in the study, the nurse-midwives were invited through WhatsApp, email and phone calls. A purposive sample of sixteen women who were at the waiting bay and staying with their spouses, were issued with letters inviting their male partners to attend FGDs in the clinic. A sample size of sixteen men was deemed sufficient to form two male FGDs. Six and seven men turned up for the FGDs on day one and two respectively.

**Data collection and management:** data collection was done using five FGDs composed of 6-7 participants to identify strategies for promoting male partner participation in MNCH services. Due to Coronavirus disease (COVID-19) epidemic, two FGDs were held virtually while three FGDs were conducted face to face in a hospital conference room. Participants in the virtual FGDs were requested to register using their official names while joining the Zoom link. Participants were reimbursed money used to purchase bundles. The discussions were held in English except for some participants in the Men FGDs who were allowed to express in Kiswahili. For participants who spoke in Kiswahili, the principal researcher being a bilingual moderator, ensured data quality was not compromised. Data collection took place between December 2021 and January 2022. The principal researcher moderated all the FGDs which were audio recorded and lasted 60-90 minutes. Participants were assigned numbers for anonymity and guidance during the discussions. Before starting the discussion, the moderator made it known to participants to respect the opinions of others and only allow one to speak at a time. During the discussions, the principal researcher made side notes for immediate reflection which helped in further clarifications from participants. Probing questions were used to seek clarity and gather more information. To ensure active participation, inactive members were called by their numbers to provide their perspectives.

**Data analysis:** first the principal researcher transcribed verbatim the audio recordings. While doing so, each voice of participants was tagged. The researcher then read through the transcripts while listening to the recordings for spelling and error checking and inserting punctuations. Later, the transcribed data was edited to remove stammers and repetition. This was done at the convenient time after each FGD. Data from the FGDs were imported into MAXQDA version 2022 for content analysis to yield themes. Both inductive and deductive coding was used. First, data from the first FGD was read and re-read for further familiarization. Initial and subsequent new codes were generated after reading data. Codes were assigned to data. General refining of codes continued as analysis proceeded to subsequent FGDs and emerging sub-categories and categories generated. Finally, analysis of the generated categories was done and conclusion drawn. Data Validity was ensured through member checking of the transcribed data and generated themes. Furthermore, the principal researcher shared the audio-recorded FGDs, transcripts and analysed data with co-researchers and their inputs incorporated in the final analysis.

**Ethical considerations:** research ethical approval and permit were sought from Jomo Kenyatta University of Agriculture and Technology Research Ethics Committee (Ref. no. JKU/2/4/896B) and National Commission for Science, Technology and Innovation (NACOSTI)- Ref. no. 207751 respectively. Consent process was established by providing information about the study and participants voluntarily requested to confirm their availability a few days prior to data collection. Confidentiality was ensured by storing voice recorded information in a password protected folder that could only be accessed by the principal researcher. Furthermore, anonymity was ensured by assigning personal identification numbers to study participants responses.

## Results

**Socio-demographic characteristics:** nearly all the nurse-midwives working in the MCH department who participated in the study were female (eighteen out of nineteen). Most of the participants (n=13) had attained diploma in Kenya Registered Community Health Nursing as the highest level of education. The findings also showed that majority of the male respondents (nine out of thirteen) were casual workers. Table 1 shows the socio-demographics of the participants.

**Table 1:** socio-demographic characteristics of participants

Nurse-midwives (n=19)			Men respondents (n=13)
**Gender**	Male	1	13
	Female	18	0
**Age**	20-30 years	4	3
	31-40 years	5	8
	> 40 years	10	2
**Marital Status**	Single	5	2
	Married	12	11
	Divorced/Separated	2	0
**Education Level**	Primary	0	3
	Secondary	0	8
	College	15	2
	University	4	0
**Employment status**	Casual workers	0	9
	Employed	19	2
	Self-employed	0	2
**Working Experience**	1-3 years	3	3
	4-6 years	11	8
	7-10 years	3	2
	> 10 years	2	0

**Strategies to promote male partner involvement:** eight categories emerged as strategies required to encourage male partner participation in MNCH services. These were: health education and sensitization training, engaging men in the MCH department, reducing waiting time, provide health services that address male health needs, invite male partners to the MCH clinic, encourage MCH staff to be receptive to couple, male partner incentivization, re-schedule MCH working days while extending work hours.

**Health education and sensitization training:** participants opined that the community as well as hospital staff needed to be sensitized on the need to promote male involvement in MNCH and reproductive services. This would demystify MCH services as being solely a woman's responsibility.

"*Am thinking now that we have religious and cultural issues that hinder male partners from getting involved, we need to engage church leaders and other community opinion leaders and inform them on the importance of male involvement in MNCH services*" (Nurse-midwife 6, FGD 1).

"*We should empower the women and inform them on the need to encourage their partners to accompany them to clinic*" (Nurse-midwife 1, FGD 2).

"*The government should send healthcare workers to the community where men will get the information. It is hard to tell a man to go to the hospital because they will think of being tested HIV*" (Male participant 2, FGD 1)

"*Change of attitude should be to all hospital workers right from the gate, cleaners, doctors and nurses. If we change attitude, we become positive about men accompanying their spouses to MCH clinic and we shall therefore warmly welcome them*" (Nurse-midwife 3, FGD 3).

**Engaging men in the MCH department:** actively engaging men while in the MCH was pointed out as an important strategy. For instance, some respondents felt that there is need to allow men to enter examination room with their spouses and allow them to participate in the process.

"*If men go to the room and the doctor engages them in the care and management, men will realize the importance of coming to the clinic*." (Nurse-midwife 1, FGD 1).

"*Involve male partners actively by letting them in during ANC. Let him listen to the fetal heart rate. That way they will feel involved. When they bring the baby for CWC, they may not be able to undress the baby but let them hold the baby during immunization*" (Nurse-midwife 5, FGD 3)

Other participants felt the need to introduce television programs and magazines in the MCH clinic to avoid boredom.

"*There should be a magazine and a television at the waiting bay to avoid men from getting bored*" (Male participant 4, FGD 2)

"*The environment/setting of the MCH clinic should be in such a way that it attracts men to be there. Having two seats for the couple, and a television set is important*" (Male participant 6, FGD 1)

**Reduce waiting time:** participants recommended on the need to attend to clients and more so couples in a relatively shorter time duration without delays as it was felt that men are generally impatient. In particular, a good number of participants proposed the need to give priority to couples and generally reduce the turnaround time to avoid men staying longer in the clinics.

"*Giving priority to couples remains a good way of encouraging male partners attend MCH clinic because it reduces waiting time*" (Male participant 7, FGD 1).

"*We can try and come up with ways of minimizing delays. Any woman accompanied with their partner should be given priority to avoid men spending a lot of time in the facility*" (Nurse-midwife 1, FGD 2).

"*The first thing is we improve on turnaround time. When they come, we should not keep them waiting for long because obviously they will get bored and go. They should be served as fast as possible*." (Nurse-midwife 4, FGD 3).

**Provision of health services that address male health needs:** the discussion gathered that health care workers need to expand the scope of MCH services to include those that address male health needs. Specifically mentioned were taking of blood pressure, body weight and prostate cancer screening for the accompanying male partners.

"*We need to be doing some things like taking blood pressure, physical examination, showing that we don't only have the interest of the woman but also the interest of the man in the family*" (Nurse-midwife 6, FGD 1).

"*We need to tailor make services that will make men be attracted to the MCH and even change the name from MCH and include male partner as well. We should also have services such as prostate cancer screening that men know will address their needs*" (Nurse-midwife 1, FGD 2).

**Issuing of male invitation letters:** to reach men in the community, it was suggested that nurse-midwives working in the MCH should issue male invitation letters to their female partners attending ANC services.

"*Actually, we should consider giving invitation letters to ANC mothers when they attend MCH services*" (Nurse-midwife 5, FGD 2).

"*I realized it is not that men don't want to go to clinics but some of them don't know that the health facility is also there to serve them. Reach out to men by inviting them to attend MCH clinic and intensify recruitment*" (Nurse-midwife 4-FGD 3).

"*The doctor should write a letter inviting the husband to accompany the wife to the MCH clinic otherwise my job supervisor cannot trust a verbal message*" (Male participant 3, FGD 1).

**Encouraging MCH staff and other hospital workers to be receptive to couples:** in order to encourage male partner accompaniment of their spouses, participants recommended a change of attitude from hospital workers including the nurse-midwives working in the MCH. This would encompass nurse-midwives being welcoming, kind, respectful and providing an extra seat for the companion.

"*Change of attitude should be to all hospital workers right from the gate, cleaners, doctors and nurses. If we change attitude towards it, we become positive about men coming to the hospital and welcome them*" (Nurse-midwife 2, FGD 3)

"*If we improve our services by being friendlier, this can encourage men*" (Nurse-midwife 7, FGD 3)

**Re-scheduling of MCH working days and extension of hours:** previous study findings showed that most men are usually busy and find no time to accompany their spouses to MCH clinic. In order to accommodate these men, participants suggested that the MCH should be opened all days including weekends and the opening days be extended to late evening.

"*The clinic is not on Saturday or Sunday and mostly opens until 2.00pm. If it was Saturday or Sunday, I may go say at 2.00pm after working*", (Male Participant 1, FGD 2).

"*Getting permission from work is the problem Yet the Clinic is open from Monday to Friday from morning to around 2.00pm. If you come after that, you are told to go the next day. It would be better if they can open on the MCH clinic beyond 2pm and weekends*", (Male Participant 6, FGD 2).

**Male partner incentivization:** participants expressed concerns about men lacking transport fare to accompany their spouses to MCH clinic due to lack of a stable income. To aid this, it was felt that offering support in terms of transport fare reimbursements would encourage male partners attend MCH clinic.

"*Men don't like wasting time especially with affairs that don't have money. If they were paid some money, it would encourage them to attend clinic but mere talking doesn't help*" (Nurse-midwife 6, FGD 3).

"*I may not have enough money to pay for the transport fare of the two of us to the clinic and unless I am supported, I will have to go out to hustle for the family*" (Male participant 3, FGD 2).

## Discussion

This study theoretically adds new knowledge on the strategies for promoting male partner involvement in MCH in low-income and middle-income countries. Lack of health education, traditional gender roles and norms appear to be a major hinderance to men's participation in MCH matters. Furthermore, men's perception of superiority makes it difficult for wives to pass the message from health providers to their husbands [10]. In this study, participants proposed the need to create awareness and sensitize the individuals, staff and the community on aspects of male partner involvement in MNCH services. Previous research interventions largely adopted community-based strategies that improved the knowledge of community members on the role and benefits of male partner involvement [9,11].

A systematic review revealed that facility-based couples' education at antenatal clinics, community-based education for men and women, workplace-based education for expectant fathers, family and community education (home visits and public meetings), social mobilisation campaign targeting husbands, midwives, and other community members can be utilized to sensitize and mobilize the community on male partner involvement in MNCH [9]. In order to effectively challenge gender roles inequity related to male accompanying their spouses to MCH clinic and being actively involved, its critical to engage boys and men early in their life in the community and clinic settings [12] and use male champions and gatekeepers who are better positioned to educate their fellow men [10].

Participants recommended for a sensitization training to change the attitude of staff working in the MCH so that they could encourage and embrace male partner MCH attendance. The findings concur with other studies which observed that there is need for sensitization training and dialoguing for heath service providers to interest them in encouraging women to go to clinics with their male partners, and to educate them on how to integrate male-friendly services in MCH service provision [12-14].

Incentivization which involve the creation of expectations of rewards if the target populations agree to practice expected behaviours has been adopted in other settings [15]. Incentivization consists of giving resources (monetary and non-monetary) to men and women to motivate them to seek MCH services in twosomes. The incentives given to men are not only perceived as motivation rewards but also as compensation for the time lost, distance traveled and other inconveniences. Studies in Uganda revealed that health care workers offered incentives such as prioritization for women accompanied by a male partner, free male specific health services, certificates for couple HIV testing, partner invitation letters, rescheduling clinic days and hours extension and creation of family support groups [16,17].

Participants proposed the need to provide health services such as blood pressure and weight checking that target male partners, transport reimbursement to needy couples and fast-tracking services to women attending ANC services with their partners. Furthermore, male participants felt that there was need for MCH staff to reschedule working days and extend hours of work in order to encourage male partner accompaniment. This would include extending working days to Saturday and work hours beyond 2pm. This strategy has effectively been applied in Uganda and Democratic Republic of Congo where working days and hours have been rescheduled when men are presumed available to access services as clients or partners [17]. Persuasion of men through invitation letters or information slips given to wives to send persuasive information that convince men to accompany their partners when seeking healthcare at clinics has proven to be successful [18].

Participants in this study suggested the need to invite men to the MCH clinic through a letter written by the hospital staff and issued to their female spouses. However, concerns were raised about those women who may have separated with their spouses or staying far away from each other. Whereas interventions such as using coercion, restriction or incentivization have been tried in other social contexts, they are more likely to result in short-term and negative outcomes because of context heterogeneities. Women have been forced to hire men “*fake husbands*” so as to skip the queue and get the services they need or otherwise be denied the MCH services, when coerced to bring partners in contexts where men are not willing to get involved. Denying services to women who do not go to clinics with partners or prioritizing couples at clinics was shown to reduce uptake of MNCH services [6-18].

## Conclusion

This study established a variety of strategies that can be adopted to encourage men to accompany their spouses to MNCH clinic. These strategies include creation of community awareness and sensitization training of staff on the importance of male partner accompaniment and involvement in MNCH services, and restructuring of the MNCH environment. Each of the male intervention strategies is necessary for behavioural change, but adoption of acceptable and appropriate multi-level strategies may sufficiently cause effectiveness in behavioural change that will result to increased levels of male partners accompanying their spouses and actively being involved in MNCH services.

### What is known about this topic

- *The levels of male partner accompanying their spouses to MNCH remains low in most sub-Saharan countries;*
- *Evidence has shown great benefits when male partners accompany and support their spouses in MNCH services;*
- *Various strategies aimed at promoting male partner involvement in MNCH have been proposed and tested in some countries*.

### What this study adds

- *It includes the perspectives of men on ways to promote their participation and involvement in MNCH services;*
- *More than one strategy may be needed to promote male partner accompaniment and involvement in MNCH services;*
- *Observes that while adopting any intervention, there is need to consider socio-cultural context of the community*.
